# Diversification of the Islamic stock market, Bitcoin, and Bullions in response to the Russia-Ukraine conflict and the COVID-19 outbreak

**DOI:** 10.1016/j.heliyon.2023.e19023

**Published:** 2023-08-07

**Authors:** Sumaira Ashraf, António Manuel Martins de Almeida, Iram Naz, Rashid Latief

**Affiliations:** a Department of Economics and Management, University of Padua, Italy; b CEFAGE Research Center, University of Évora, Portugal; c CITUR Research Center, University of Madeira, Portugal; d Military College of Signals, National University of Science & Technology, Pakistan; e School of Finance, Xuzhou University of Technology, China

**Keywords:** Islamic stock market, Bullion, Bitcoin, Dynamic connectedness, COVID-19 pandemic, Russia-Ukraine war, TVP-VAR, Portfolio diversification, Hedging, Safe haven, Financial assets

## Abstract

This study investigates the interconnectedness of the Islamic stock market, Bullion, and Bitcoin as diversifiers for portfolios, exploring their role as hedges and safe havens. The analysis covers the period from January 2015 to December 2022, with a particular focus on the influence of the COVID-19 pandemic and the Russia-Ukraine War on the MSCI World Islamic Index, bullions (Gold, Silver, Platinum, Nickel, Palladium, and Aluminium), and Bitcoin, employing a time-varying parameter vector autoregression (TVP-VAR) model. During crisis periods, our findings reveal that the transmission and reception of shocks among these assets varied, with a heightened level of co-movement observed during the pandemic and war periods. These results emphasise the importance of considering the dynamic nature of financial assets' connectedness in asset investment decisions, particularly in times of crisis. Furthermore, the findings suggest that Bullion can serve as a hedge for both Bitcoin and the Islamic stock market. The study also explores the optimal diversification of investment portfolios and highlights the importance of adhering to Islamic principles in portfolio diversification. By integrating Islamic rules into the diversification process, investors can enhance the effectiveness and relevance of their investment strategies.

## 1 Introduction

Investment portfolios require effective diversification to mitigate financial market volatility. To achieve this goal, investors match negatively correlated securities within their portfolios. As a result, precious metals, such as gold and silver, which have a negative correlation with stock market returns, have become a popular diversification tool for risk-averse investors [1]. Additionally, the Islamic equity market has gained attention as a way to recover from the 2008 global financial crisis and become an effective diversification tool in financial markets [2]. Recently, cryptocurrency has also emerged as a financial tool for portfolio risk minimisation. Bitcoin is one of the largest and most popular digital currencies, with peer-to-peer transactions without intermediaries [3].

Most Islamic financial tools have been developed to attract the funds of investors looking for a Sharia-compliant investible universe. Islamic stocks are traded on a faith-based system in accordance with the tenets of Shariah-based Islamic laws [4]. This solution allows religious investors to invest without contradicting their beliefs [5]. Islamic stock markets attract investors through five key principles: no usury, risk sharing, restricted trading, a specified contract, and operations following Sharia rules [6]. Commodities that comply with these rules provide interest-free transactions and serve as essential portfolio diversification tools [7].

Islamic stocks, or Shariah-compliant stocks, are stocks of companies whose activities and operations are compatible with Islamic principles and values. These may include companies in industries such as healthcare, education, and renewable energy, and must avoid businesses that are considered forbidden (haram), such as gambling, tobacco, and alcohol. Precious metals like gold and silver are considered suitable for Muslim investors as they have intrinsic value and are widely accepted as a medium of exchange by Sharia law. According to Islamic legal opinions (Fatwas), Bitcoin is also permissible under Sharia law as long as it is used for legitimate purposes and not for speculation or fraudulent activities. In our study, we focus on the need of investors for less risky sharia-compliant financial assets such as the Islamic stock market (MSCI World Islamic Index - MSWI), Bitcoin, and Bullions, including Gold, Silver, Platinum, Nickel, Palladium, and Aluminium.

The mainstream studies in the literature have explored the diversification benefits of alternative portfolio investment tools. Bullion markets, especially gold, silver, and platinum, have gained investors' attention along with the Islamic stock markets [8]. Spot trading in the commodity market is also permissible under the tenets of Sharia law, making precious metals an additional important financial instrument for those who prefer to invest according to Islamic rules. Many researchers have tried to prove the hedging benefit of gold, the most important precious metal in national and local portfolios. Such studies found gold to be a vital portfolio diversification tool [[9], [10], [11], [12]], and similar findings were observed for other precious metals, including silver and platinum [[13], [14], [15]]. [16,17] compared Bitcoin to gold and found similarities. Furthermore, researchers have evidenced Bitcoin as a profitable investment [[18], [19], [20], [21]]. Studies have shown that gold and other precious metals can provide a hedge and safe haven against volatility in cryptocurrencies and Islamic stocks [[22], [23], [24], [25], [26], [27]]. However, interestingly, none of these studies has explored the relationship between the Islamic stock market, Bullion, and Bitcoin.

The COVID-19 health crisis caused drastic liquidity and a global economic collapse [28]. The Russia-Ukraine conflict caused worldwide disruption after the health crisis. Recent studies have reported the negative impacts on the financial markets, commodity markets, and energy prices [[29], [30], [31], [32]]. Some studies have also reported the dynamic connectedness among major financial markets, different commodity markets, and cryptocurrencies [12,[33], [34], [35], [36], [37]]. However, none of the previous studies has compared the effects of the Covid-19 pandemic and the Russia-Ukrainian war for alternative financial assets and considered the hedging role of Bullions for other financial assets.

Our study contributes to the existing literature in four ways: first, we examine the three alternative investment portfolio diversifiers, namely the Islamic Stock Market, Bitcoin, and Bullions (Gold, Silver, Platinum, Nickel, Palladium, and Aluminium), to identify the optimal diversifier for an investment. Second, we conduct a comprehensive analysis of the dynamic connectedness between them using the time-varying parameter vector auto-regression (TVP-VAR) method analysing generalized impulse response functions (GIRF) and generalized forecast error variance decompositions (GFEVD) for measuring the connectedness. Third, we compare the results of the connectedness between two crisis periods, namely the COVID-19 pandemic and the Russia-Ukraine war. Fourth, we investigate the role of Bullion as a hedge and safe haven for Bitcoin and MSWI, as well as the impact of the two crisis periods on their returns.

Our overall findings suggest that the role of the assets in transmitting and receiving shocks varied during different periods of crisis. The results indicate that the financial assets were not independent during the pandemic and war periods, and their co-movement increased. The role of the assets as a net transmitter or receiver was influenced by the crisis events, such as the COVID-19 pandemic and the Russia-Ukraine War, with some assets becoming more prominent as transmitters of shocks while others as receivers. This research is also the first to report that Bullion can play an important role in reducing the overall risk of an investment portfolio during different periods of crisis. The findings highlight the importance of considering the dynamic nature of the connectedness of financial assets in investment decisions, especially during periods of crisis. The result of this study provides important insights for investors who seek to diversify their portfolios in line with Islamic principles and can be used as a reference for investors who want to make informed and timely investment decisions.

The remainder of our work is structured as follows: Section two reviews the existing studies on the Islamic Stock Market, Bitcoin Market, and Bullion Markets; Section three describes our sample and empirical methodology; Section four presents the empirical findings of the study; and Section five ends with the conclusion and recommendations.

## 2 Literature review

Previous research has primarily focused on the hedging and diversification benefit of the bullion market and its role in Islamic stock markets, as well as the role of Bitcoin as an investment asset. Additionally, the literature has compared Islamic and traditional finance markets. However, there is limited analysis directly examining the role of Bullion in the Islamic stock market, and there is a lack of exploration of portfolio diversification opportunities among Islamic stocks, Bullion, and bitcoin during crisis periods.

In the last decade, there has been a recognised demand for Sharia-compliant portfolio diversification tools to attract the accumulated oil wealth of Islamic countries within the history of Islamic finance. Islamic stocks have unique features such as ethical investment goals, screening of ratios, avoidance of interest-bearing loans, and restrictions on structured financial products (e.g., derivatives), which differentiate them from conventional stocks [38]. Islamic stock indices and Islamic mutual funds are also considered more profitable than their traditional counterparts due to the additional financial screening criteria applied to the companies [39,40].

Previous studies have compared the performance of Islamic and conventional financial markets, investigated portfolio diversification benefits, and examined co-movements with other financial assets. For example [41], compared the performance of twenty-four global and regional exchange market indices and found that Islamic indices performed better than conventional stock indices [42]. compared the performance of twenty-two different stock markets and proved that the Islamic markets had higher performance than mainstream markets during the financial crisis. More recently [43], analyzed the time-varying risk spillover between large stock exchanges in the USA, Japan, Europe, and Asia and precious metals (gold, silver, palladium, and platinum) using the Spillover Index developed by Ref. [44]. They found evidence of volatility spread between precious metals and Islamic stock markets [45]. provided evidence that both Islamic equity and stock markets are effective portfolio diversifiers for green bonds. Furthermore [46], suggested that emerging Asian investors may use Islamic equities as portfolio diversification assets against Bitcoin, particularly since Bitcoin prices experience large upward trends.

The studies on Bullion have predominantly focused on gold as a hedging and diversification tool. Some researchers, including [11,[47], [48], [49]] have suggested that gold is an effective hedging and portfolio diversification tool and may serve as a safe haven during times of stress and turmoil in American stock markets, major European stock markets (Germany, UK), and emerging markets (China, India). However, there is a need for more studies on the application of gold as a hedge or safe heaven, as well as exploration of other precious metals as substitute assets for investment. For instance Ref. [1], found that precious metals, including gold, platinum, and silver, are excellent diversifiers and hedging tools in the American stock markets [25,50]. found that gold is a superior hedging tool compared to platinum and silver and offers more hedging benefits during inflationary and normal periods. On the other hand [13], suggest that silver, platinum, and palladium act as havens, while gold does not. Additionally [8,50], also demonstrate that platinum is an excellent hedging and portfolio diversification tool.

Bitcoin, a digital currency that emerged after the global financial crisis, has gained significant attention and interest in economics literature due to its remarkable growth in size and market capitalisation. It is a new generation of currency that became more popular in a climate of fear as people lost confidence in fiat currency, which lacked backup security [17]. Cryptocurrencies, including Bitcoin, may offer diversification benefits relative to other financial assets, including stock returns [51]. [52] examined the co-movement between Bitcoin and Islamic assets, including Sukuk and Islamic Stock index, and found that the portfolio diversification benefits of Bitcoin and Islamic assets vary across time and frequencies [53,54]. also concluded that Bitcoin qualifies as a safe haven against downturns in Islamic stock markets.

The previous literature primarily studied the role of precious metals as a hedge, safe haven, or diversifier with the conventional (non-Islamic) stock markets. Only a few studies have extended this research to Islamic stock markets in line with the tenets of Islamic law. For example [4], found a long-term relationship between gold prices, oil, and the FTSE Bursa Malaysia Emas Shariah Index, while [55] showed strong co-movement between Islamic share markets and commodity markets. Furthermore [2], explored the long-term relationship between precious metals (including gold, silver, platinum, and palladium) and the Islamic stock markets of thirty-two different countries and found that gold and palladium were effective diversifiers for developing countries, while all four precious metals were effective diversifiers for developed Islamic stock markets.

[23] recently investigated the dynamic spillover impacts of West Texas Intermediate (WTI) oil and the S&P 500 stock index before and during the COVID-19 pandemic. They used gold and Bitcoin (BTC) prices and found that only gold had a sheltering role for oil and stock index returns during the pandemic [56]. recently provided evidence on the dependence structure, connectedness, and spillover among green bonds and other key financial markets, including energy stocks, world stocks, and volatility indices, in the context of COVID-19. Their study used the [44] spillover approach, wavelet multiple and cross-correlation analysis, and data spanning from 2016 to 2020. The results showed that the correlation pattern among green bonds and other studied markets significantly decreased from short to long-term, which was consistent with the findings by Ref. [57]. [58] found that the world stock index transmitted the highest spillover to other markets in net terms, while corporate bonds were net receivers of spillover from the studied markets [31]. observed a correlation between US Islamic Stock, gold, and Bitcoin, suggesting that gold is a safe hedging tool for diversification, except during the COVID-19 pandemic and the Russia-Ukraine wartime period. The study also found that Bitcoin is more volatile than gold. Similarly [12], found that gold works as a strong hedge for the USA, the UK, Italy, Spain, Germany, France, Russia, and China, and Bitcoin is a strong hedge in the USA and acts both as a strong hedge and safe haven in China.

In recent years, studies have demonstrated that precious metals can serve as a hedge and safe haven against volatility in cryptocurrencies and Islamic stocks. [23,24], and [59] have provided evidence of the positive impact of precious metals on cryptocurrencies. [22,25,26,60], and [46] have found evidence of the positive impact of precious metals on Islamic stocks, especially during periods of economic uncertainty. These studies suggest that investors should consider diversifying their portfolios by including precious metals with cryptocurrencies or Islamic stocks to reduce overall risk.

While previous studies have focused on Bitcoin, gold, and stock markets, none has explored the relationship between precious metals, the Islamic stock market, and Bitcoin during crisis periods. The present study aims to contribute to the existing body of knowledge by investigating the dynamic connectedness among these three markets, exploring the role of precious metals as a hedge and safe haven, and examining the impact of major events such as the COVID-19 pandemic and the Russia-Ukraine war on their returns. This analysis aims to establish a new time-varying pattern that could manage the connectedness among Bullion, Bitcoin, and Islamic stock markets. Furthermore, this study aims to enhance understanding of the similarities and dissimilarities, both inter and intra, with respect to information spillovers.

## 3 Data description and methodology

### 3.1 Data

This study aims to evaluate the effectiveness of bullions (Gold, Silver, Platinum, Nickel, Palladium, and Aluminium), cryptocurrency (Bitcoin), and the Islamic stock market (MSCI World Islamic Index) as diversification tools during financial turmoil. The study examines the changing interdependence between these assets during the Russia-Ukraine War and the COVID-19 pandemic. The data used in this study includes daily closing future prices of these assets from January 01, 2015, to December 31, 2022. This time frame was selected to ensure adequate coverage of Bitcoin data and to capture the behaviour of the assets during the crisis periods.

Additionally, the ERS unit root test of [61] was used to check for the stationarity of the raw time series data. If the data is non-stationary, it needs to be transformed to obtain a stationary series before further analysis can be performed. In this case, the first log differences ln (xit) − ln (xit−1) were taken, which can be interpreted as index returns. This transformation helps to stabilize the variance and remove any trends or seasonality in the data, allowing for more accurate statistical analysis.

Table 1 reports descriptive statistics of the log-returns of the examined bullions, bitcoin, and Islamic stock market. The results show that Bitcoin has the highest return, while platinum has the lowest return. However, Bitcoin also has the highest variance, indicating a significant difference in returns when compared to other investment instruments. The findings indicate that the series is significantly non-normally distributed (as evidenced by the [[62], [63], [64]] tests) and stationary at the 1% significance level.

**Table 1:** Summary statistics.

	BT	MSWI	GLD	SLV	PT	NI	PD	AI
Mean	0.203*	0.016	0.022	0.022	−0.005	0.036	0.042	0.013
Variance	22.296***	0.935***	0.882***	3.281***	2.850***	8.623***	5.251***	1.641***
Skewness	−0.877***	−1.018***	−0.137**	−0.539***	−0.375***	2.520***	−0.812***	0.003
Kurtosis	10.324***	14.667***	4.458***	6.324***	5.865***	148.257***	12.194***	2.732***
JB	8915.174***	17825.057***	1621.786***	3345.317***	2841.635***	1788856.252***	12301.258***	606.541***
ERS	−2.745***	−3.877***	−5.214***	−5.417***	−13.810***	−11.650***	−19.314***	−10.493***
Q (20)	13.540	75.183***	13.337	21.576***	27.671***	95.529***	31.744***	13.722
Q^2^ (20)	62.317***	1320.020***	209.277***	289.466***	440.682***	1128.253***	341.878***	523.410***
Unconditional correlations								
BT	1.000***							
MSWI	0.217***	1.000***						
GLD	0.075***	0.121***	1.000***					
SLV	0.101***	0.266***	0.780***	1.000***				
PT	0.134***	0.396***	0.551***	0.609***	1.000***			
NI	0.047**	0.175***	0.186***	0.233***	0.249***	1.000***		
PD	0.110***	0.347***	0.358***	0.425***	0.530***	0.269***	1.000***	
AI	0.084***	0.256***	0.146***	0.214***	0.240***	0.241***	0.253***	1.000***

Notes: The table displays summary statistics for Bitcoin, the MSCI World Islamic Index, and bullions (GLD, SLV, PT, NI, PD, AI); ***, **, and * denote significance levels of 1%, 5%, and 10%, respectively; Skewness: D'Agostino skewness test; Kurtosis: Anscombe-Glynn test; JB: Jarque-Berra normality test; ERS [61]: unit-root test; *Q*(20) and *Q*^2^(20) [65]: weighted portmanteau test.

Additionally, the data show pronounced autocorrelation in both the series and the squared series [65], which implies that each series' mean and variance vary over time. Thus, utilising a TVP-VAR model with a time-varying variance-covariance structure appears to be an appropriate econometric framework to capture all these factors. The unconditional correlation matrix showed the strongest correlation between gold and silver and the weakest between bitcoin and nickel.

### 3.2 Methodology

The main objective of this paper is to examine the dynamic interconnectedness between Bitcoin, the Islamic stock market (represented by the MSCI World Islamic Index), and precious metals (Gold, Silver, Platinum, Nickel, Palladium, and Aluminium) during two crisis periods: the COVID-19 pandemic and the Russia-Ukraine War. The results of the analysis will be useful for investors who aim to effectively diversify their investment portfolios to mitigate the impact of financial market volatility. To do this, the paper uses the TVP-VAR approach proposed by Refs. [66,67], which is based on [68,69].

The TVP-VAR based on the Bayesian Information Criterium (BIC) that is estimated in this paper is defined in Eqs. (1), (2) below:

(1) $$y_{t}=A_{t}y_{t-1}+u_{t}\;u_{t}|F_{t-1}\;∼\;Ν\left(0,V_{t}\right)$$

(2) $$vec\left(A_{t}\right)=vec\left(A_{t-1}\right)+v_{t}\;v_{t}|F_{t-1}∼Ν\left(0,S_{t}\right)$$

where $F_{t-1}$ represents the information available till t-1, $y_{t}$, $y_{t-1}\text{,}$ and $u_{t}$ is defined as *m × 1* (*k* is the data sample size) dimensional vector and A_*t*_ and V_*t*_ are defined as m × m dimensional matrices. Moreover, $vec\left(A_{t}\right)$ and $v_{t}$ are defined as m^2^ × 1 dimensional vectors, while $S_{t}$ is m^2^ × m^2^ dimensional matrix.

TVP-VAR approach itself is insufficient and requires a connectedness approach between variables which is dependent upon time-varying parameters and error variances. For this purpose, two parameters, i.e., GIRF-generalized impulse response functions [70] and GFEVD-generalized forecast error variance decompositions [71]. Computing these parameters requires transformation from TVP-VAR to its vector moving average (TVP-VMA) using the equality expressed in Eq. (3):

(3) $$z_{t}=\sum_{i=1}^{k}A_{it}y_{t-i}+u_{t}=\sum_{j=0}^{\infty }A_{j,t}u_{t-j}$$

The advantage of using GIRF, represented by $\psi_{j,t}\left(K\right)$, for K cast horizon, is that it is robust in interpreting VAR models due to its independence over the order of errors. GIRF captures the difference of dynamics among and between all variables and is mathematically defined in Eq. (4) as:

(4) $$GIRF\left(K,\sqrt{H_{jj,t}},F_{t-1}\right)=E\left(y_{t+k}|\in_{j,t}=\sqrt{H_{jj,t}},F_{t-1}\right)-E\left(y_{t+J}|F_{t-1}\right)$$

$$\psi_{j,t}\left(K\right)=H_{jj,t}^{\frac{-1}{2}}\Lambda_{K,t}H_{t}\in_{j,t}$$

Following, GFEVD contributes to presenting the contribution of each variable in terms of the forecast error variance of a variable. In other words, how much does the forecast variance of one variable impact the forecast error variances of other variables. It is mathematically defined in Eq. (5) as:

(5) $$\omega_{ij,t}\left(K\right)=\frac{\sum_{t=1}^{K-1}\psi_{ij,t}^{2}}{\sum_{j=1}^{m}\sum_{t=1}^{K-1}\psi_{ij,t}^{2}}$$

with $\sum_{j=1}^{m}\psi_{ij,t}\left(K\right)$ = 1, $\sum_{i,j=1}^{m}\left(\psi_{j,t}\left(K\right)\right)$ = m. The connectedness measures derived from GFEVD were derived as follows:

(6) $${TO}_{jt}=\sum_{i=1,i\neq j}^{m}\psi_{ij,t}\left(K\right)$$

(7) $${FROM}_{jt}=\sum_{i=1,i\neq j}^{m}\psi_{ji,t}\left(K\right)$$

(8) $${{{NET}_{jt}=TO}_{jt}-FROM}_{jt}$$

(9) $${TCI}_{t}^{g}\left(K\right)=\sum_{i,j=1,i\neq j}^{m}\psi_{ij,t}^{g}\left(K\right)$$

(10) $${NPDC}_{ij,t}=\psi_{ji,t}\left(K\right)-\psi_{ij,t}\left(K\right)$$

(11) $${PCI}_{ijt}\left(K\right)=2\left(\frac{\psi_{ij,t}^{g}\left(K\right)+\psi_{ji,t}^{g}\left(K\right)}{\psi_{ii,t}^{g}\left(K\right)+\psi_{ij,t}^{g}\left(K\right)+\psi_{ji,t}^{g}\left(K\right)+\psi_{ij,t}^{g}\left(K\right)}\right)$$

As ${\varphi^{\prime }}_{ij,t}^{g}$ (*H*) represents the impact of a shock of variable *j* on i. Eq. (6) defines the aggregated impact of a shock on variable *j* from all the remaining variables (total connectedness), while Eq. (7) defines the aggregated influence of all variables on *j* (*total directed connectedness from others* on *j*). Subtracting (Eq. (7)) from (Eq. (6)) provides a net total directional connectedness, depicting whether *j* is a net receiver or transmitter of the shock (Eq. (8)). Followed by Eq. (9) is the total connectedness index which illustrates the impact of *j* on other variables. It is worth mentioning that all connectedness measures the aggregated impact, while Eqs. (10), (11) presents net pairwise directional connectedness that defines a bilateral relationship between two variables and pairwise connectedness index between two variables (*i* and *j*).

The volatility of financial markets caused by various crises has heightened the uncertainty of international investments. This uncertainty has persisted in recent years, exacerbated by the economic turmoil caused by the COVID-19 pandemic and the Russia-Ukraine War. In this context, it becomes challenging for investors to find a Sharia-compliant investment universe. The diversification opportunities offered by Islamic markets and cryptocurrencies have also weakened. These events have prompted international investors to search for new financial markets, such as Bullion, which is also considered permissible under Sharia law. These investors aim to attain a significant degree of portfolio risk diversity without resorting to financial markets that contravene their beliefs. Financial analysts and portfolio managers also aim to design optimal portfolios incorporating a mix of financial assets with varying volatility levels. We tested the safe heaven properties of Bullions against Bitcoin and MSWI, as Bullion tends to hold its value or appreciate during times of market stress or uncertainty. This is due to their scarcity and intrinsic value as physical commodities, which can make them less susceptible to fluctuations in currency value or changes in supply and demand. By including these assets in a portfolio, investors may be able to mitigate risk and potentially achieve better returns.

## 4 Empirical results

### 4.1 Connectedness analysis based on the TVP-VAR approach

Table 2 displays the average dynamic connectedness between Bitcoin (BT), the MSCI World Islamic Index (MSWI), Gold (GLD), Silver (SLV), Platinum (PT), Nickel (NI), Palladium (PD), and Aluminium (AI) returns. The main diagonal represents the impact of shocks on their own variance, while the off-diagonal elements indicate the influence of one asset on the others and vice versa (TO). The research divided the sample period into three parts to study the change in interdependence during Pre-Crisis, COVID-19 pandemic, and Russia-Ukraine War period.

**Table 2:** Average dynamic connectedness table.

Pre-Crisis (2015/01/01–2020/01/12)									
	BT	MSWI	GLD	SLV	PT	NI	PD	AI	FROM
BT	95.56	0.62	1.05	0.41	0.33	0.58	0.78	0.67	4.44
MSWI	0.82	68.94	1.49	4.06	5.73	7.66	6.22	5.08	31.06
GLD	0.53	0.83	47.77	29.54	14.93	1.53	4.03	0.84	52.23
SLV	0.21	2.35	26.77	43.31	14.54	4.23	6.13	2.46	56.69
PT	0.16	3.82	14.76	16.12	48.69	4.45	9.53	2.46	51.31
NI	0.35	6.95	1.9	5.79	5.68	62.51	5.22	11.59	37.49
PD	0.45	5.63	4.74	8.22	11.72	5.08	60.95	3.22	39.05
AI	0.87	5	1.4	3.89	3.63	12.58	3.77	68.86	31.14
TO	3.39	25.21	52.12	68.02	56.55	36.12	35.69	26.32	303.41
NET	−1.05	−5.85	−0.11	11.33	5.24	−1.37	−3.36	−4.82	37.93
COVID Outbreak (2020/01/13–2022/02/23)									
	**BT**	**MSWI**	**GLD**	**SLV**	**PT**	**NI**	**PD**	**AI**	**FROM**
BT	71.86	8.95	2.24	3.20	6.26	2.34	4.42	0.73	28.14
MSWI	6.60	49.45	3.89	5.74	11.58	8.98	9.64	4.12	50.55
GLD	1.87	2.89	45.00	27.01	14.43	1.71	5.59	1.50	55.00
SLV	2.37	4.45	23.85	39.66	16.73	3.20	7.71	2.04	60.34
PT	3.76	8.90	12.99	16.44	38.21	5.32	11.87	2.50	61.79
NI	2.05	10.41	2.26	4.88	8.06	56.27	4.27	11.81	43.73
PD	3.51	9.92	7.09	9.13	15.56	3.45	49.03	2.30	50.97
AI	0.89	5.68	1.75	3.20	5.14	13.91	2.80	66.63	33.37
TO others	21.05	51.20	54.06	69.60	77.75	38.92	46.30	25.01	383.90
NET	−7.09	0.65	−0.93	9.25	15.97	−4.82	−4.67	−8.36	47.99
Russia-Ukraine Conflict (2022/02/24–2022/12/31)									
	**BT**	**MSWI**	**GLD**	**SLV**	**PT**	**NI**	**PD**	**AI**	**FROM**
BT	67.90	22.56	1.21	1.66	1.88	0.74	0.98	3.08	32.10
MSWI	19.12	56.16	2.69	5.94	8.46	0.39	3.00	4.24	43.84
GLD	1.26	3.26	36.03	22.79	13.77	6.59	12.33	3.96	63.97
SLV	2.27	4.70	23.81	36.19	14.17	5.31	10.01	3.53	63.81
PT	1.58	6.58	15.06	14.78	38.26	3.93	14.86	4.96	61.74
NI	1.11	0.68	11.51	8.81	6.27	60.15	8.89	2.57	39.85
PD	1.34	4.05	14.50	11.08	15.80	6.06	41.08	6.10	58.92
AI	4.40	9.72	6.55	5.70	7.10	5.77	8.66	52.10	47.90
TO others	31.08	51.56	75.34	70.76	67.45	28.78	58.72	28.44	412.13
NET	−1.02	7.72	11.37	6.95	5.71	−11.07	−0.20	−19.46	51.52

Notes: This table presents the average directional connectedness between the returns of Bitcoin, the MSCI World Islamic Index, and bullions in three different periods: full sample, the COVID-19 outbreak, and the Russia-Ukraine War.

Over the full sample period, the assets had moderate co-movement, with 37.93% of the forecast error variance (GFEVD) in one asset being attributed to innovations in all others. During the crises, the co-movement increased, with the total connectedness index (TCI) reaching 47.99% and 51.52% during COVID-19 and the Russia-Ukraine War, respectively. This result showed that the assets were not independent during the crisis periods. During the COVID-19 pandemic, PT, SLV, and MSWI were the main sources of shocks. However, after the onset of the Russia-Ukraine War, the magnitude of PT and SLV decreased while MSWI increased. GLD's role changed dramatically from a shock receiver during the pandemic to a shock transmitter during the war, potentially due to inflation fears leading to a demand for safe-haven investments and an EU ban on Russian gold imports.

**Fig. 1:**
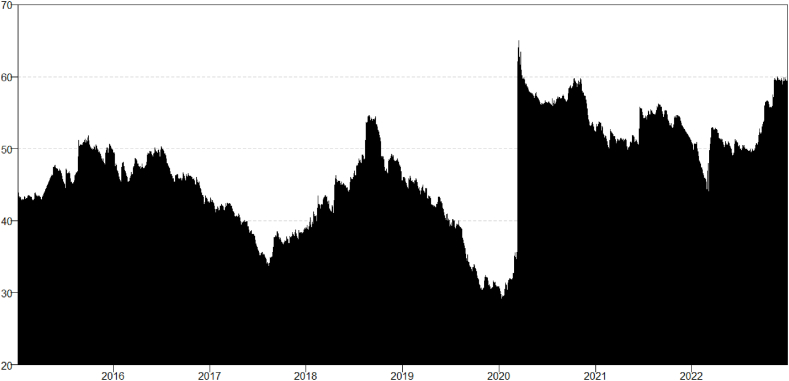
Dynamic total connectedness (TCI).

The results presented in Table 2 are the aggregate results that consider the total study period. To determine whether the connectedness across financial assets varied over time and how it was affected by the pandemic and war period, we estimate the dynamic total connectedness index (TCI). The results in Fig. 1 indicate that the financial assets were not independent of each other during the pandemic and war periods. The TCI reached exceptional heights during these crises, suggesting increased co-movement and reduced diversification among the financial assets studied, such as Bullion, Bitcoin, and the Islamic market. The increase in TCI could be due to negative investor sentiment, reduced economic activity, and increased demand for safe-haven investments during these crisis periods [72].

**Fig. 2:**
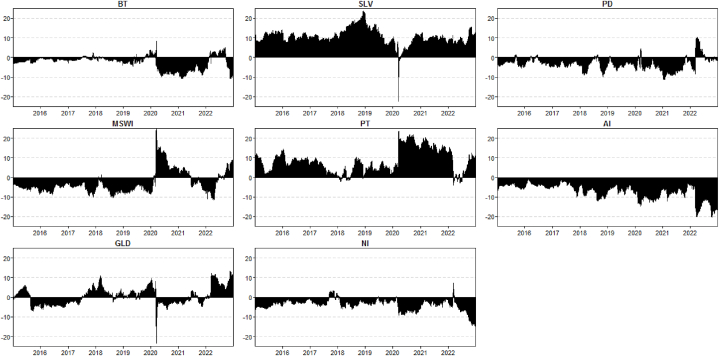
Net total directional connectedness for all assets: Bitcoin (BT), Islamic markets (MSCI) and Bullions (GLD, SLV, PT, NI, PD, AI).

Turning our attention to the net total directional connectedness (Fig. 2), which allows us to determine the role of one asset in the network over time. The plot shows that SLV and PT are the main transmitters of shock during the sample period. For GLD, it is hard to summarise as a net transmitter and receiver; it was a net transmitter before the pandemic and during the war period, whereas it was a net receiver during the pandemic period. Both SLV and GLD depict a sharp decline in volatility during the start of the pandemic. Further, AI is the net receiver during the whole sample period. A similar receiving pattern can be viewed in NI and PD, except for a smaller period of the Russia-Ukraine conflict, where they became net transmitters. We further show that BT and MSWI were the volatility receivers before the pandemic and war, whereas both became the sender of stress during the pandemic and war periods.

**Fig. 3:**
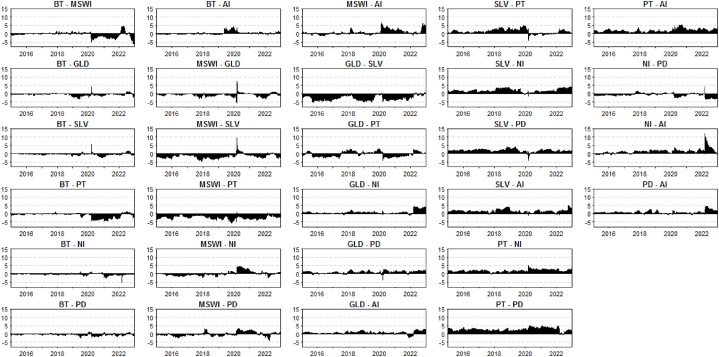
Net pairwise directional connectedness between all pairs of financial assets: Bitcoin (BT), Islamic stock market (MSWI) and Bullions (GLD, SLV, PT, NI, PD, AI).

Fig. 3, the results of the study suggest that the net pairwise directional connectedness of spillovers across pairs of bitcoin, MSWI, and bullions is greater during periods of pandemic and war. Bitcoin and MSWI became net transmitters of shocks during these periods, whereas bullions experienced an increase in the magnitude of volatility spillovers. The findings highlight the importance of considering financial market conditions when analysing the behaviour of different financial assets and their interdependence.

**Fig. 4:**
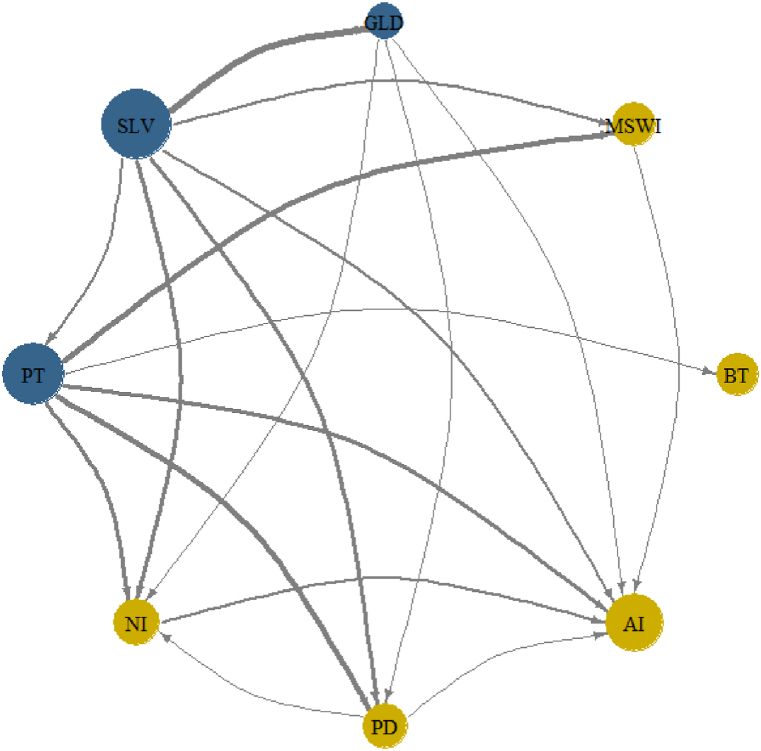
Network of net pairwise directional connectedness index (NPDC). The blue nodes in the graph denote the transmitters (receivers) of NPDC, meaning that they are most likely to influence other assets in the network. The node size represents this node's net connectedness effect, with larger nodes indicating a greater connectedness effect. The arrows in the graph indicate the direction of NPDC, and the width of the lines denotes the connectedness intensity.

Additionally, Fig. 4 supports the findings in Fig. 3 and demonstrates the heightened connectedness during pandemic and war periods. The graph highlights the increased directional connectedness among the assets and highlights the main transmitters and receivers. The visual representation of the NPDC helps to better understand the direction and strength of the relationships among different financial assets, providing insights into how they may interact in times of market stress.

### 4.2 Portfolio performance evaluations

Using the estimation results of our model, we assess the hedge effectiveness of MSWI and Bitcoin in bivariate minimum variance portfolios (MVP) constructed with bullions. The statistics of the optimal portfolio weights and the hedging effectiveness (HE) for all the bivariate portfolios over the full sample period (January 2015 to December 2022) are presented in Table 3. The asset weight for MSWI/Bullions portfolios is higher (ranging from 53% to 95%) compared to Bitcoin/Bullions portfolios (ranging from 3% to 19%). Our findings indicate that the hedging effectiveness of all bullions with MSWI is statistically significant but not very strong (ranging from 7% to 50%). Conversely, the hedging effectiveness of SLV, PT, NI, and PD is higher (ranging from 81% to 88%) and statistically significant with Bitcoin. These results suggest that investors should allocate fewer investments in Bitcoin and more in Bullions to achieve a portfolio with minimum variance.

**Table 3:** Summary statistics for optimal portfolio weights.

	Mean	Std.Dev.	5%	95%	HE
Panel A: Bitcoin/Bullions					
BT/GLD	0.03	0.02	0.00	0.08	0.96
BT/SLV	0.11	0.06	0.03	0.22	0.87***
BT/PT	0.08	0.04	0.02	0.16	0.88*
BT/NI	0.19	0.18	0.03	0.66	0.82***
BT/PD	0.16	0.09	0.03	0.32	0.81***
BT/AI	0.05	0.03	0.00	0.10	0.93
Panel B: MSWI/Bullions					
MSWI/GLD	0.53	0.11	0.30	0.70	0.50***
MSWI/SLV	0.85	0.09	0.70	0.96	0.12***
MSWI/PT	0.85	0.08	0.73	1.00	0.07***
MSWI/NI	0.91	0.16	0.47	1.00	0.13***
MSWI/PD	0.95	0.03	0.91	1.00	0.03***
MSWI/AI	0.69	0.21	0.11	0.90	0.35***

### 4.3 The impact of the COVID-19 pandemic and the Russia-Ukraine war

The COVID-19 pandemic and Russia-Ukraine War have contributed to heightened uncertainty and volatility in financial markets, thereby increasing the associated risk with different financial assets. To gain a deeper understanding of the impact of these events on portfolio performance, we conducted a comprehensive analysis of Bullion's ability to serve as a hedge against Bitcoin and Islamic stocks during the pandemic and war period. Specifically, we calculated the optimal portfolio weights and the hedging effectiveness index for these periods and compared the results with the full sample period.

(12) $$\Delta HE_{C}=HE_{COVID-19}–\;HE_{Full\;period}$$

(13) $$\Delta HE_{W}=HE_{WAR}\;–\;HE_{Full\;period}$$

The ΔHE_C_ and ΔHE_W_ indices in Eqs. (12), (13) were used to assess the difference in hedging effectiveness during the pandemic and war period and the full sample period. A positive value for ΔHEC and ΔHEW indicates a beneficial hedging strategy during the pandemic and war period, respectively.

Table 4 represents the findings, which indicate that the hedging effectiveness of Bitcoin with bullions was not statistically significant, except for palladium during the pandemic. Additionally, the results reveal a sudden decline in the hedging effectiveness of Bitcoin with bullions during the war. This decline can be attributed to the uncertainty affecting most precious metals produced in Russia, which are crucial for the supply chain of modern manufacturing. Only nickel and palladium provide hedging effectiveness with Bitcoin. In contrast, the findings suggest that the bullion market serves as a stronger hedge for the Islamic stock market during the war and pandemic. This is evidenced by the comparatively less significant decrease in hedging effectiveness when comparing the MSWI and bullion portfolios. Consequently, investors may find it advantageous to allocate more investments to MSWI and Bullion to construct optimal portfolios with minimum variance. These findings align with the conclusions of [26], who determined that precious metals, particularly gold, can act as safe havens and diversifiers for Islamic stocks during periods of economic uncertainty. Overall, our research suggests that investing in Bullion, particularly in conjunction with Islamic stocks, can provide a more stable hedge against financial turmoil compared to investing solely in Bitcoin.

**Table 4:** COVID-19 and Russia-Ukraine war effects.

	COVID-19 pandemic			Russia-Ukraine War		
Panel A: Bitcoin/Bullions						
	ѡ	HE	ΔHE_C_	Ѡ	HE	ΔHE_W_
BT/GLD	0.02	0.95	−0.01	0.04	0.94	−0.02
BT/SLV	0.14	0.79	−0.08	0.14	0.79	−0.08
BT/PT	0.08	0.80	−0.08	0.15	0.78	−0.10
BT/NI	0.05	0.90	0.08	0.75	0.30***	−0.52
BT/PD	0.15	0.74**	−0.07	0.40	0.56***	−0.25
BT/AI	0.03	0.94	0.01	0.10	0.79	−0.14
Panel B: MSWI/Bullions						
MSWI/GLD	0.49	0.50***	0.00	0.37	0.51***	0.01
MSWI/SLV	0.90	0.11***	−0.01	0.81	0.08***	−0.04
MSWI/PT	0.95	0.03***	−0.04	0.87	0.04***	−0.03
MSWI/NI	0.70	0.28***	0.15	0.97	0.03***	−0.10
MSWI/PD	0.97	0.02***	−0.01	0.96	0.01***	−0.02
MSWI/AI	0.48	0.59***	0.24	0.77	0.09***	−0.26

Note: This table reports the optimal portfolio weights and differences in hedging effectiveness (HE) during the due to COVID-19 pandemic and Russia-Ukraine War. ѡ is the optimal portfolio weight. HE is the hedging effectiveness index, and ***, **, and * denote their significance levels at 1%, 5%, and 10%, respectively. ΔHE_C_ measures the difference in HE values between the COVID-19 pandemic period and the full sample period, and ΔHE_W_ measures the difference in HE values between the Russia-Ukraine War period and the full sample period. The higher the HE value, the greater the risk reduction, and vice versa.

## 5 Conclusion and recommendation

This study examines the effectiveness of various assets, including bullions (Gold, Silver, Platinum, Nickel, Aluminium, and Palladium), cryptocurrency (Bitcoin), and the Islamic stock market (MSCI World Islamic Index) as diversification tools during financial turmoil. The research analyses the changing interdependence among these assets during the Russia-Ukraine War and the COVID-19 pandemic, using daily data from January 01, 2015, to December 31, 2022, and employing a time-varying parameter vector autoregression (TVP-VAR) model. The findings of this study suggest that Bullion, cryptocurrency, and the Islamic stock market can serve as useful portfolio tools during periods of financial turmoil, exhibiting negative correlations over the long term. However, their interdependence increased during the COVID-19 pandemic and the Russia-Ukraine War, indicating that they were not independent of each other during these crisis periods. Moreover, the results indicate that bullions, particularly GLD, SLV, and PT, were the primary transmitters of shocks to the Islamic stock market and Bitcoin during both crisis periods. The direction of spillovers suggests that Bullion could serve as a safe haven for investors during times of uncertainty, as evidenced by the increased demand for bullions during the pandemic and war.

Furthermore, the study employs a minimum variance portfolio (MVP) approach to estimate the optimal portfolio weights and hedging effectiveness of the Islamic stock market and Bitcoin when combined with bullions. The findings suggest that investors should allocate less to Bitcoin and more to bullions to achieve minimum variance in their portfolios.

The results of this study have significant implications for investors, policymakers, and regulators. For investors, the analysis demonstrates that bullions such as Gold, Silver, Platinum, Nickel, Aluminium, Palladium, the Islamic stock market and Bitcoin can effectively diversify portfolios during financial crises. These findings are particularly relevant to investors looking for Sharia-compliant investment opportunities and aiming to safeguard their portfolios against market risk. Bullion can be used as a hedge for both Bitcoin and the Islamic stock market, with higher diversification benefits observed for the latter.

Policymakers should take note of the importance of promoting the use of Bullion as an alternative investment asset in times of financial uncertainty. Central banks and policymakers responsible for managing monetary policy and ensuring financial stability should consider the potential impact of changes in bullion demand on the broader financial system. Governments can create a favourable environment for bullion investment and incentivise portfolio diversification. These results are also significant for policymakers aiming to develop Sharia-compliant financial products and markets, as they may need to encourage the growth of bullion markets and other permissible alternative investment assets.

Regulators play a crucial role in maintaining the stability and integrity of financial markets. The findings of this study can inform regulatory policies by encouraging market participants to invest in Bullion. The negative correlations between bullions and cryptocurrency suggest that bullions can be used as a hedge against potential risks associated with cryptocurrency investments. Regulators should carefully balance investor protection with the promotion of innovation and growth in the cryptocurrency market. Additionally, regulators can support Sharia-compliant investment tools by providing more guidance and oversight.

While this study focuses on diversification between traditional bullions, a single Islamic market, and Bitcoin, future research could explore diversification tools to optimise portfolios involving Islamic markets and other cryptocurrencies such as Zcash, ripple, and DASH.

## Author contribution statement

Sumaira Ashraf: Conceived and designed the analysis; Analyzed and interpreted the data; Contributed analysis tools or data; Wrote the paper.

Antonio Manuel Martins de Almeida: Analyzed and interpreted the data; Wrote the paper.

Iram Naz: Analyzed and interpreted the data; Wrote the paper.

Rashid Latief: Analyzed and interpreted the data; Wrote the paper.

## Funding statement

Sumaira Ashraf acknowledges the financial support of 10.13039/501100001871Fundação para a Ciência e a Tecnologia (grant UIDB/04007/2020).

## Data availability statement

The data that has been used is confidential.

## Declaration of competing interest

The authors declare that they have no known competing financial interests or personal relationships that could have appeared to influence the work reported in this paper.
